# Mitral paravalvular abscess with left ventriculo-atrial fistula in a patient on dialysis

**DOI:** 10.1186/1749-8090-4-35

**Published:** 2009-07-16

**Authors:** Tadashi Kitamura, James Edwards, Suchi Khurana, Robert G Stuklis

**Affiliations:** 1 Department of Cardiothoracic Surgery, Royal Adelaide Hospital, North Terrace, Adelaide SA 5000, Australia; 2 Department of Cardiology, Royal Adelaide Hospital, North Terrace, Adelaide SA 5000, Australia

## Abstract

**Background:**

Infective endocarditis in hemodialysis patients is challenging but is becoming more common recently.

**Case report:**

A 64-year-old man with end-stage renal disease on hemodialysis presented with infective endocarditis of mitral valve and coronary artery disease after commencing training for home hemodialysis. During a course of antibiotic treatment the patient developed left ventriculo-atrial fistula due to mitral paravalvular abscess. Abscess debridement followed by reconstruction of the mitral annulus with fresh autologous pericardial patch and mitral valve replacement using a mechanical prosthesis with concomitant coronary artery bypass grafting was performed successfully.

**Conclusion:**

Timely diagnosis, proper antibiotic treatment and early surgical intervention including aggressive debridement should improve the outcome of this high-risk disease.

## Introduction

The end-stage renal disease is becoming more common recently and so is infective endocarditis (IE) in hemodialysis (HD) patients. Accordingly, surgeons have been encountering challenging situations to overcome this high-risk disease more often. We present a successfully treated case with IE complicated by left ventriculo-atrial fistula due to mitral paravalvular abscess in an HD patient with concomitant coronary artery disease.

## Case presentation

**Figure 1:**
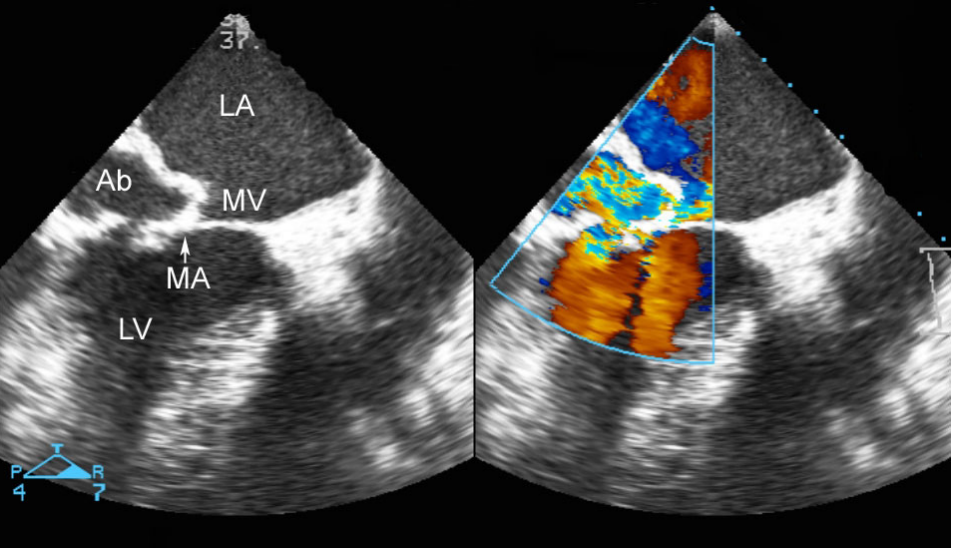
**Transesophageal echocardiogram showing mitral paravalvular abscess with ventriculo-atrial fistula**. LA indicates left atrium; MV, mitral valve; MA, mitral annulus; LV, left ventricle; and Ab, abscess.

**Figure 2:**
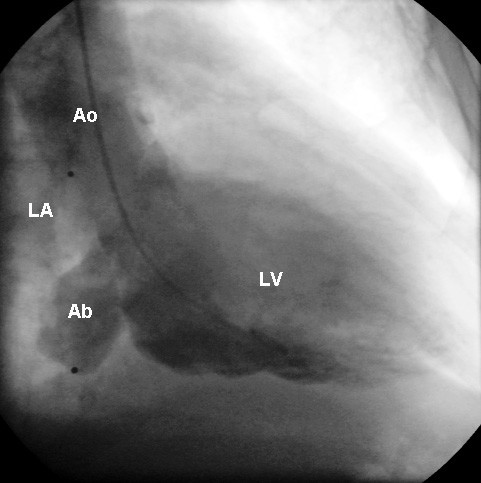
**Left ventriculogram showing severe mitral regurgitation with paravalvular abscess**. LA indicates left atrium; LV, left ventricle; Ab, Abscess; and Ao, aorta.

**Figure 3:**
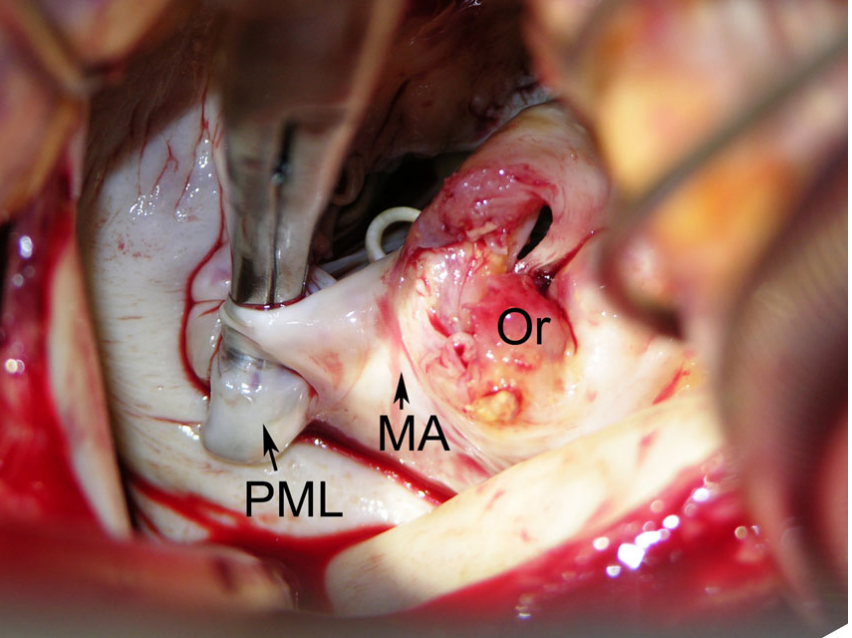
**Abscess cavity opening into left atrium**. PML indicates posterior mitral leaflet; MA, mitral annulus; and Or, orifice of abscess.

**Figure 4:**
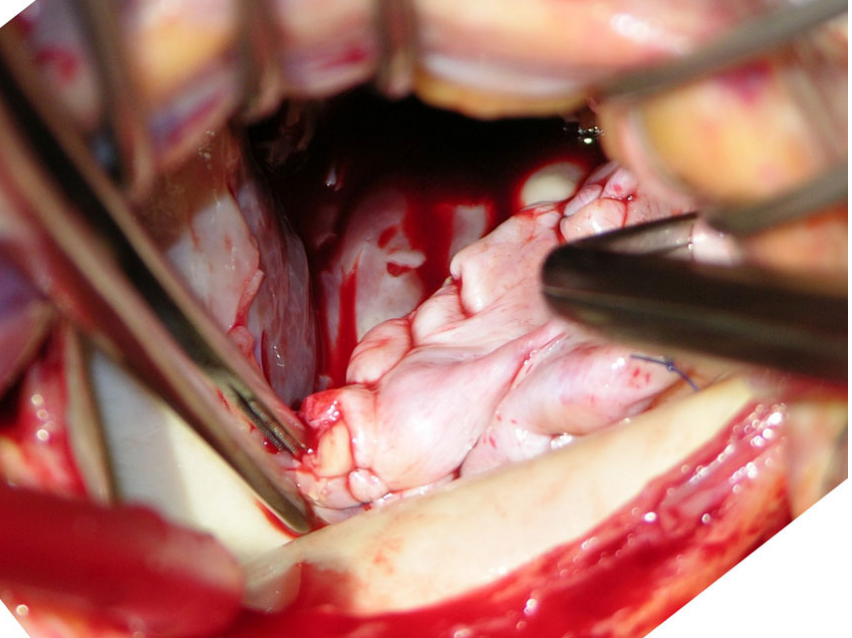
**Reconstruction of the mitral annulus with autologous pericardial patch**.

A 64-year-old man with end-stage renal disease on HD due to chronic glomerulonephritis presented with a 2-day history of lethargy after commencing training for home HD. Echocardiography revealed vegetation on the posterior mitral leaflet with trivial mitral regurgitation and blood cultures confirmed *Staphylococcus aureus*. During the course of antibiotic treatment including benzylpenicillin the patient developed sudden shortness of breath with New York Heart Association functional class III. Twelve-lead electrocardiogram showed sinus rhythm with first-degree atrioventricular block. Transesophageal echocardiography (Figure 1) and left ventriculography (Figure 2) demonstrated severe mitral regurgitation with a cavity posterior to the mitral annulus connecting to both left ventricle and left atrium. Coronary angiography revealed 90% stenosis in the left anterior descending artery and complete occlusion of the proximal right coronary artery with diffusely diseased downstream collateralized from the left coronary artery. Antibiotic treatment was continued for further two weeks and the patient underwent surgery after finishing a proper course of antibiotics. Operative findings included destroyed posterior mitral leaflet with an abscess extending underneath the mitral annulus, opening into the left atrium (Figure 3). The patient underwent abscess debridement followed by reconstruction of the mitral annulus with fresh autologous pericardial patch (Figure 4) and mitral valve replacement using a mechanical prosthesis with concomitant left internal mammary artery graft to the left anterior descending artery. Histopathology of the valve showed acute neutrophilic inflammation but it was culture-negative. Postoperatively the patient recovered well without any signs of reinfection, paravalvular leak or ECG change.

## Discussion

The number of the patients with end-stage renal disease who are on HD is increasing every year and it is well known that IE in HD is significantly more common. The potential explanations for the increased incidence of IE in HD patients are; increased incidence of degenerative heart valve disease with accelerated development of valvular calcification related to abnormal calcium-phosphorus homeostasis, high incidence of bacteremia due to vascular access, and impaired immune system because of metabolic abnormalities [1]. Home HD is a very useful method to improve the patient's quality of life and can be performed very safely as long as the patient is appropriately trained [2], but it should be noted that in this particular case the patient developed IE immediately after commencement of training for self-cannulation.

HD is associated with a high incidence of IE especially in mitral position and it also increases the risk of following surgical treatment [3]. When it is complicated by a paravalvular abscess, it becomes even more challenging. It has been reported that patients who had surgery tended to survive more than those who did not [4]. It has to be taken into account that a number of patients on HD with IE are too sick to have surgery, contributing to higher mortality for patients without having surgery. However, there is no doubt that significant hemodynamic deterioration caused by IE has to be treated surgically and that all efforts must be made to perform surgery in a better condition. Although it is better served with surgical intervention after proper antibiotic treatment [5], mitral paravalvular abscess sometimes requires surgery in the active state due to fistula or pseudoaneurysm formation [6]. Fortunately, in our case, we could wait till antibiotic treatment finished even with left ventriculo-atrial fistula. The most important principle of surgical treatment for IE is to reduce risk of reinfection, and aggressive debridement is required to achieve this. However, patients on HD often have annular calcification and extensive debridement of such cases can increase the risk of postoperative paravalvular leak after valve replacement. Autologous pericardium has been used with good long-term results for reconstruction of the mitral annulus to secure the prosthetic valve and to prevent postoperative paravalvular leak after mitral valve replacement with an uneven annulus [7].

Patients on HD also tend to have high incidence of coronary artery disease and concomitant coronary artery surgery at the time of valve surgery for IE makes the risk even higher. Coronary angiography should be performed whenever possible to evaluate the risk precisely before the operation.

## Conclusion

IE in HD patients is more common recently but it is still associated with very high mortality especially when complicated by paravalvular abscess and other comorbidities including coronary artery disease. Timely diagnosis, proper antibiotic treatment and early surgical intervention including aggressive debridement should improve the outcome, as was demonstrated by our case.

## Consent

Written informed consent was obtained from the patient for publication of this case report and accompanying images. A copy of the written consent is available for review by the Editor-in-chief of this journal.

## Competing interests

The authors declare that they have no competing interests.

## Authors' contributions

All authors contributed equally to the manuscript and all authors read and approved the final manuscript.
